# Chvostek sign

**DOI:** 10.1002/ccr3.2373

**Published:** 2019-08-16

**Authors:** Roberto Rodrigues, Luís Teles

**Affiliations:** ^1^ Serviço de Atendimento Urgente Centro de Saúde de Machico, ACES RAM, SESARAM E.P.E Madeira Portugal

**Keywords:** acute hypocalcemia, Chvostek sign, emergency medicine, total thyroidectomy

## Abstract

Careful anamnesis can act as gasometry in services with few resources. In this clinical case, a detailed clinical history made it possible to suspect the presence of acute hypocalcemia, a biochemical anomaly after confirmed in gasometry. Acute hypocalcemia can be life threatening, necessitating urgent treatment. Sometimes it can be managed with oral ambulatory treatment.

A 43‐year‐old woman presents to Emergency Department complaining of extremities paresthesias, tremor, and muscle cramps which started two days before. A total thyroidectomy undergone 4 days before was the only relevant past medical history.

Which gasometry alteration can be expected, in the presence of the physical sign shown in the video S1?

The gasometry confirmed hypocalcemia (Ca^2+^ = 0.72 mmol/L). The patient was discharged with oral calcitriol and calcium carbonate. A month later, she was completely asymptomatic, after restarted oral treatment during follow‐up.

The disruption of parathyroid gland function due to total thyroidectomy is the most common cause of acute hypocalcemia.^1^

## CONFLICT OF INTEREST

None to declare.

## AUTHOR CONTRIBUTIONS

Both authors have made substantial contributions to the design, data collection, and interpretation of the data. Both authors participated in the writing or critical review of the article regarding intellectually important content. The main author has reviewed the final version of the manuscript and approved its publication.
